# Author Correction: Co-option of epidermal cells enables touch sensing

**DOI:** 10.1038/s41556-023-01326-2

**Published:** 2023-12-06

**Authors:** Federica Mangione, Joshua Titlow, Catherine Maclachlan, Michel Gho, Ilan Davis, Lucy Collinson, Nicolas Tapon

**Affiliations:** 1 Apoptosis and Proliferation Control Laboratory, The Francis Crick Institute, London, UK; 2 Department of Biochemistry, University of Oxford, Oxford, UK; 3 Electron Microscopy Science Technology Platform, The Francis Crick Institute, London, UK; 4 Sorbonne Université, CNRS, Laboratoire de Biologie du Développement, Institut de Biologie Paris Seine (LBD-IBPS), Paris, France

**Keywords:** Morphogenesis, Cell biology

Correction to: *Nature Cell Biology* 10.1038/s41556-023-01110-2, published online 23 March 2023.

In the version of the article originally published, Fig. 2d contained an error, for which we apologize. The images in the time series were inadvertently derived from two different experiments due to an error during the assembly of the panels. We have now provided a corrected version of this panel with all frames coming from the same time series. This error does not affect any of the results or conclusions in the article. The original and corrected Fig. 2d are shown below in Fig. 1. The figure has now been amended in the HTML and PDF versions of the article.

**Fig. 1:**
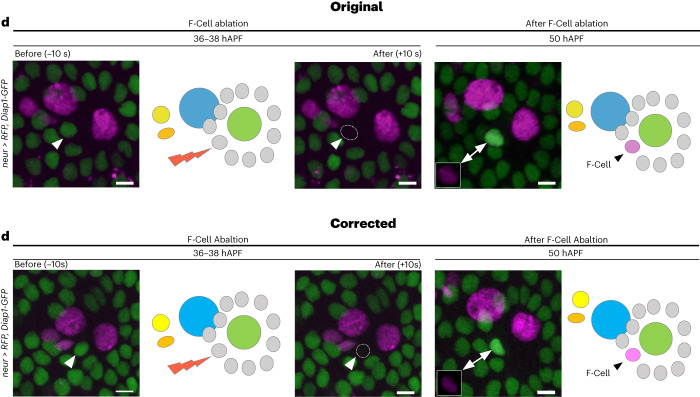
**Original and revised Fig. 2d.**

