# Supplementary material for: Co-option of epidermal cells enables touch sensing
Source: Nat Cell Biol. 2023 Mar 23;25(4):540–9. doi: 10.1038/s41556-023-01110-2 (PMC10104782; doi:10.1038/s41556-023-01110-2)
Supplement: Supplementary file 1 [file 41556_2023_1110_MOESM1_ESM.pdf]

nature portfolio

Corresponding author(s): Federica Mangione and Nicolas Tapon

Last updated by author(s): Jan 26, 2023

Reporting Summary

Nature Portfolio wishes to improve the reproducibility of the work that we publish. This form provides structure for consistency and transparency in reporting. For further information on Nature Portfolio policies, see our Editorial Policies and the Editorial Policy Checklist.

Statistics

For all statistical analyses, confirm that the following items are present in the figure legend, table legend, main text, or Methods section.

n/a	Confirmed	
☐	☒	The exact sample size (*n*) for each experimental group/condition, given as a discrete number and unit of measurement
☒	☐	A statement on whether measurements were taken from distinct samples or whether the same sample was measured repeatedly
☐	☒	The statistical test(s) used AND whether they are one- or two-sided<br>*Only common tests should be described solely by name; describe more complex techniques in the Methods section.*
☒	☐	A description of all covariates tested
☐	☒	A description of any assumptions or corrections, such as tests of normality and adjustment for multiple comparisons
☐	☒	A full description of the statistical parameters including central tendency (e.g. means) or other basic estimates (e.g. regression coefficient) AND variation (e.g. standard deviation) or associated estimates of uncertainty (e.g. confidence intervals)
☐	☒	For null hypothesis testing, the test statistic (e.g. *F*, *t*, *r*) with confidence intervals, effect sizes, degrees of freedom and *P* value noted<br>*Give P values as exact values whenever suitable.*
☒	☐	For Bayesian analysis, information on the choice of priors and Markov chain Monte Carlo settings
☒	☐	For hierarchical and complex designs, identification of the appropriate level for tests and full reporting of outcomes
☒	☐	Estimates of effect sizes (e.g. Cohen's *d*, Pearson's *r*), indicating how they were calculated

Our web collection on statistics for biologists contains articles on many of the points above.

Software and code

Policy information about availability of computer code

Data collection	For electrophysiology recordings, data were collected with MultiClamp700B amplifier and Axon Digidata 1550A A/D board (Molecular Devices). Microscopy data (Laser Scanning Microscopy and Electron Microscopy) were acquired using the Zen software (Zeiss LSM 880, LSM780 and Sigma VP SEM, ZEN 2.1). SEM images were acquired with a JEOL Microscope (JCM-6000Plus).
Data analysis	GraphPad Prism 9 was used for data representation and the PAST 4.04 software was used for statistical analyses. Images were analyzed using Fiji and image segmentation was performed using the TrakEM2 plugin of Fiji. Electrophysiology recordings were analyzed off line with the Clampfit software v10.7 (Molecular Devices). Animations of Volume EM data were made with Blender (software v2,9). Adobe Photoshop 2022 and Adobe Illustrator 2022 were used for figure assembly and diagrams. No custom data analysis code was required in this study.

For manuscripts utilizing custom algorithms or software that are central to the research but not yet described in published literature, software must be made available to editors and reviewers. We strongly encourage code deposition in a community repository (e.g. GitHub). See the Nature Portfolio guidelines for submitting code & software for further information.

Data

Policy information about availability of data

All manuscripts must include a data availability statement. This statement should provide the following information, where applicable:

- Accession codes, unique identifiers, or web links for publicly available datasets
- A description of any restrictions on data availability
- For clinical datasets or third party data, please ensure that the statement adheres to our policy

The data supporting the findings of this study are available within this paper and associated Extended Data, Source Data and Supplementary Information. Additional information are available from the corresponding authors on reasonable request.

Field-specific reporting

Please select the one below that is the best fit for your research. If you are not sure, read the appropriate sections before making your selection.

☒ Life sciences ☐ Behavioural & social sciences ☐ Ecological, evolutionary & environmental sciences

For a reference copy of the document with all sections, see nature.com/documents/nr-reporting-summary-flat.pdf

Life sciences study design

All studies must disclose on these points even when the disclosure is negative.

Sample size	No statistical methods were used to predetermine sample size. Sample sizes were chosen on the basis of previous experience, published literature and current standards in the field (PMID: 30517870, PMID: 10744543, PMID: 27807168, PMID: 12441297). Statistical analyses were performed comparing different groups (e.g., mutants versus controls) with similar sample size. Test for normal distribution were performed before choosing the statistical test for significance to be applied in any given dataset.
Data exclusions	No data points/outliers were excluded from the analyses.
Replication	Experiments were typically replicated at least three times, unless stated otherwise, and only those experiments for which repeats gave comparable outcomes were included in the manuscript.
Randomization	Samples were allocated to experimental groups according to genotype, and appropriate controls were performed in all experiments, so randomization was not required. Adult females were used as control and experimental samples for all behavioural assays and for electrophysiology.
Blinding	The investigator were not blinded to allocation during experiments or analysis, as group allocation was clearly visible in samples due to phenotypic and/or behavioural changes.

Reporting for specific materials, systems and methods

We require information from authors about some types of materials, experimental systems and methods used in many studies. Here, indicate whether each material, system or method listed is relevant to your study. If you are not sure if a list item applies to your research, read the appropriate section before selecting a response.

Materials & experimental systems

n/a	Involved in the study	
☒	☐	Antibodies
☒	☐	Eukaryotic cell lines
☒	☐	Palaeontology and archaeology
☐	☒	Animals and other organisms
☒	☐	Human research participants
☒	☐	Clinical data
☒	☐	Dual use research of concern

Methods

n/a	Involved in the study	
☒	☐	ChIP-seq
☒	☐	Flow cytometry
☒	☐	MRI-based neuroimaging

Animals and other organisms

Policy information about studies involving animals; ARRIVE guidelines recommended for reporting animal research

Laboratory animals	Fruit flies (Drosophila melanogaster) at pupal stages (males and females) and at adult stage (females).
Wild animals	No wild animals were used in this study.
Field-collected samples	This study did not involved samples collected from the field.
Ethics oversight	No ethical approval was required. The use of Drosophila melanogaster does not require ethical approval or guidance.

Note that full information on the approval of the study protocol must also be provided in the manuscript.
